# Syphilis, *blanchiment* and French colonial medicine in sub-Saharan Africa during the interwar period

**DOI:** 10.1017/mdh.2023.29

**Published:** 2023-10

**Authors:** Guillaume Linte

**Affiliations:** Institute for Ethics, History, and the Humanities (iEH2), Centre médical universitaire, University of Geneva, 1 rue Michel Servet, CH-1211 Geneva 4, Switzerland

**Keywords:** colonisation, syphilis, health, neglected diseases, skin diseases, medicine

## Abstract

During the interwar period, France put unprecedented efforts into public health measures targeting the colonised populations of sub-Saharan Africa. This investment in health was seen as crucial to ensuring the renewal of the African labour force needed for the economic development of the colonies. Syphilis, although less deadly than other endemic or epidemic diseases such as yellow fever, sleeping sickness and bubonic plague, was one of the most widespread infections in France’s sub-Saharan colonies. This article demonstrates the contradictory nature of the colonial medicine approach to this disease during the interwar years. The negative impact of syphilis on population growth in Africa made it a major threat to the colonial project, and France put significant, costly investment into tackling the disease, focusing its efforts on maternal and child health. However, a closer look at syphilis control in sub-Saharan Africa reveals that the disease was also minimised as a public health issue, under-resourced and downplayed by colonial doctors and administrators. This neglect was embodied in the invention of a new colonial disease, ‘exotic syphilis’, which was presented as being a relatively benign skin disease among the African populations. It was also reflected in care practices, via a form of mass medicine based on the use of *blanchiment*, which consisted of knowingly limiting treatment to a superficial effect.

## Introduction

During the interwar years, unprecedented efforts went into public health within the French colonial empire. While the colonies’ health services were essentially designed for European soldiers and settlers, the development of the Assistance Médicale Indigène [Native Medical Service] (AMI) marked a turning point in the colonial project. This was a period of experimentation, such as the mass sleeping sickness control campaigns that were rolled out in the wake of the *Mission Jamot*, which began its work in the 1920s in Cameroon.^1^ Back in France, syphilis was the subject of particular scrutiny during this period, due to the threat it was seen to pose to the necessary regeneration of the nation after the devastating population loss of the First World War.^2^ In addition to the decline in births, which was attributed to miscarriages and infertility, the spectre of ‘racial degeneration’, embodied by hereditary syphilis [*hérédosyphilis*],^3^ hung over the country’s future. During the interwar period, these concerns gradually shifted to the ‘native’ populations of the colonies. In this context, however, the discourse was different: it was a matter of preserving the labour resources crucial to the development of the empire’s territory. In Africa, the objective was clear: ‘*faire du nègre*’ (i.e. promoting population growth) was necessary in order to exploit the continent’s wealth and to provide soldiers for the colonial troops.^4^ As such, public health campaigns against various regional endemic diseases and epidemics with a significant impact on the population (including death and disability), such as malaria, sleeping sickness, bubonic plague and yellow fever,^5^ were often prioritised over diseases considered to be less dangerous, such as syphilis. In many colonies, however, the ‘great pox’ was widespread and was one of the most common infectious diseases seen by physicians in the colonial health services.

The history of syphilis in a colonial context has received increasing attention from historians over the past thirty years. The case of the British Empire has been the most extensively studied, following on from Megan Vaughan’s work in the early 1990s,^6^ while recent studies have explored the history of venereal diseases in the Belgian Congo, Portuguese Guinea and the German colonies prior to the First World War.^7^ French sub-Saharan Africa was also the subject of various studies in the 2000s and 2010s, mainly centring on a single colony or locality.^8^ The most extensive data available are on Senegal, thanks to the work by Mor Ndao and Kalala Ngalamulume, who have highlighted the increasing desire of the colonial powers to take action against venereal diseases, with little success.^9^ They have also revealed the inability of the French to understand the African societies who inhabited the administered territories, resulting in the introduction of ineffective or even unworkable health policies, notably in relation to prostitution.

Based on a study of the discourses and practices associated with treating syphilis in the French colonial empire, I argue that redefining syphilis as a ‘skin disease’ served to justify its neglect by French colonial health services. This was characterised by limited attention and action in relation to the scale of the public health problem, its seriousness for individuals and its impact on society. This neglect was embodied in many different aspects, including minimising the danger of the disease, downplaying its impact, limited or targeted resources, incomplete treatment and fatalistic attitudes. It was also underpinned by a dominant medical discourse that supported and validated the colonial strategy against syphilis, despite the existence of a few dissenting voices.

Since the late twentieth century, the concept of ‘neglect’ has become essential to understanding a group of tropical diseases known as Neglected Tropical Diseases (NTDs). More recently, a subgroup of diseases with skin manifestations has been singled out within this group: the skin NTDs.^10^ The understanding of the mechanisms that produce neglected diseases has thus far been little explored as a field of study, particularly in terms of its historical dimension.^11^ It is clear that colonial doctors and administrators in the first half of the twentieth century downplayed the danger of certain diseases by presenting them as skin diseases. In this article, I show that the colonial definition of syphilis was designed precisely to make it a skin disease, in the form of an ‘exotic’ disease primarily characterised by skin manifestations and specific to African and Asian populations. I thus shed light on how the colonial period influenced the representation of skin diseases as being relatively benign and not a priority area for investment. Syphilis is a prime example of a disease whose seriousness was played down on the grounds that it was supposedly a mere skin disease.

The article focuses on several key aspects of the history of syphilis in the French colonial empire. Firstly, I show how it was redefined by medicine as a ‘benign’ skin condition among the colonised populations and go on to provide a non-exhaustive overview of the diverse range of medical representations of this disease, which was embodied in particular in the form of an ‘exotic syphilis’ supposedly less dangerous than that seen in metropolitan France. Building on this, the second part of the article looks at the impact of the discovery of the microbial agent (*Treponema pallidum*, 1905) on the definition of the disease and analyses the debate that emerged in the 1910s and 1920s about the existence of different types of syphilis, especially ‘neurotropic’ and ‘dermotropic’ forms. In the third part, I focus on a practice central to syphilis control in the French empire: *blanchiment* [lit. ‘clearing’]. This practice constituted the basis of a ‘static’ form of mass medicine (as opposed to the mobile medicine that developed in the wake of the *Mission Jamot*) that was both economical and ‘effective’ in its ability to provide the illusion of care. I then examine the debates and uncertainties that accompanied the roll-out of the colonial syphilis control programme. The criticisms directed at this programme by a minority of doctors, on the grounds of its short- and long-term iatrogenic effects, called into question both the narrative of the history of syphilis in Europe and that of the triumph of modern medicine. Finally, in the last two sections, I analyse what I define as a ‘rhetoric of neglect’, which consisted of shifting the responsibility for the failure of the syphilis control programme onto the colonised populations and fatalistically presenting them as impossible to treat. This discourse, founded on racial and cultural conceptions, dominated colonial medicine during the interwar period^12^ and made it possible to justify the use of health care practices that were considered morally questionable by some in the medical community.

## Exotic syphilis: a benign skin disease

In France, the debate about whether syphilis was a single disease entity was the subject of major research in the early twentieth century, notably in a colonial context. This was approached from various angles: (1) whether the disease was caused by a single infectious agent, (2) the diversity of symptoms based on ‘race’ and (3) differences in the symptomatology based on climate. One of the first to propose the concept of an ‘exotic syphilis’ was dermatologist Édouard Jeanselme, on his return from a study trip to the Far East.^13^ In 1902 Jeanselme began to teach at the Institut de Médecine Coloniale and gave a series of lectures there on tropical dermatology that was published in 1904 under the title *Cours de dermatologie exotique* [Lectures on Exotic Dermatology].^14^ In the decades that followed, this book became a key reference for venereologists.^15^ The eighth lecture in the course was devoted to ‘exotic syphilis’,^16^ which Jeanselme presented as being as yet little studied by his colleagues: ‘Contrary to what you might think, gentlemen, exotic syphilis is a new subject, one that has not yet been fully elucidated.’^17^

What was ‘exotic syphilis’? According to Jeanselme, while the disease was ‘pandemic’ and did not differ in nature from one place to another, it took a different form depending on the ‘race’ of the individuals who contracted it. It is notable that while the infectious agent had not yet been identified, there was no doubt in the physician’s mind that syphilis was caused by a micro-organism. Moreover, Jeanselme argued, while the climate itself did not directly influence the disease, the ‘social habits’ imposed by climate on the colonised populations gave it ‘a very particular stamp’: ‘Thus, for example, an apathetic, sober native of the inter-tropical regions will not react to the poison of syphilis in the same way as an intemperate, overexerted European.’^18^ However, he also saw syphilis as presenting in multiple forms, depending on the colony and people. Jeanselme was particularly interested in comparing the seriousness of syphilis, arguing that it was, to varying degrees, more benign among Africans and Asians than among Europeans:

The seriousness of syphilis in the tropics is controlled by various factors. The most important of these is *race.* Blacks are relatively immune to the pox; they are not wholly protected from the disease, far from it, but among them it usually takes a benign course.^19^

Jeanselme did, however, see the disease as being less benign among certain populations, such as those of Madagascar and Indochina, where it was supposedly more virulent: the ‘exotic syphilis’ affecting these regions caused ‘incurable mutilations’ as it was characterised by deep bone and skin involvement. But he still viewed it as less serious due to its limited lethality. This ‘relative benignity’, he explained, was due to the fact that ‘among coloured men, parasyphilitic affections are the exception, if not completely unknown’, as a result of their sober, indolent way of life, compared to the ‘various forms of nervous overexertion to which Westerners are all too often exposed.’^20^ Despite downplaying the seriousness of ‘exotic syphilis’ in this way, Jeanselme still sounded the alarm about the growing spread of the disease in the colonies, highlighting the risks of labour shortages and ‘racial degeneration’: ‘Untreated, the pox will bastardise the race, decrease births, and increase deaths.’^21^ This idea, around which colonial action would coalesce during the interwar period, was thus already well established in Jeanselme’s mind at the turn of the century.

While Jeanselme’s ideas were key to the history of syphilis in the first half of the twentieth century, they were also part of a long-term debate about the definition of syphilis in the tropics. It had long been confused with other exotic diseases unfamiliar to European physicians and, more specifically, with yaws – a disease with some similar skin symptoms but caused by a different subspecies of the bacterium responsible for syphilis: *Treponema pertenue.* Since the eighteenth century, it has been debated whether yaws and syphilis are two different diseases or one and the same. According to Katherine Paugh, who has focused on the British Atlantic world, after an indecisive eighteenth century, ‘By the early nineteenth century, it was the general consensus among British medical authorities that the great pox was a different malady from yaws.’^22^ However, the diagnosis of yaws and syphilis was still often confused by European doctors, as several historical studies focusing on the first half of the twentieth century have suggested.^23^ In Africa, syphilis transmission was associated with promiscuity, sometimes on a par with sexual intercourse. But for yaws, especially in children and young adults, promiscuity was also identified as one of the main modes of transmission. Confusion about symptoms was compounded by the difficulty of identifying sources of contamination.

## Civilisation and syphilisation

The question of whether syphilis was a single disease entity was not solely of interest in a colonial context but also prompted more general research in dermatology and syphilography. The identification of the treponeme responsible for the disease *(Treponema pallidum* subsp. *pallidum*) in 1905 paved the way for further biological investigations. The link between syphilis and two nervous diseases, general paralysis and tabes dorsalis, was first confirmed by Hideyo Noguchi and Joseph W. Moore in 1913 after observing *Treponema pallidum* in the brains of deceased patients.^24^ The following year, in 1914, in a note presented to the Académie des Sciences, Constantin Levaditi and Auguste Marie claimed that the treponeme responsible for general paralysis was in fact ‘a separate, neurotropic, variety of the pallidum spirochaete.’^25^ Their work was interrupted by the war, but, upon its resumption in 1919 they confirmed the existence of different species of *Treponema pallidum*,^26^ on the basis of extensive animal – and human – experiments.^27^ More specifically, they described the existence of two different forms of syphilis, linked to two separate infectious agents or ‘viruses’: (1) a ‘dermotropic’ virus, which manifested mainly in bone and skin involvement, and (2) a ‘neurotropic’ virus, which affected the nervous system and resulted in general paralysis. The two strains should not, however, be considered equal: they argued that the dermotropic strain was the original version of the treponeme, while the neurotropic strain was the product of selective evolution. In Levaditi and Marie’s demonstration, the colonial world served as a comparative model for understanding the original strain of syphilis. Their biological approach shed new light on Jeanselme’s earlier observations: the supposed differences observed between metropolitan France and the colonies could now be explained by the unequal distribution of the two strains.^28^ This hypothesis did not, however, exclude a racial dimension. Why was it that only metropolitan France was affected by the ‘neurotropic virus’, while the dermotropic version of the disease reigned supreme in the rest of the empire? This is where the selective, evolutionary nature of the treponeme came into play:

Indeed, it is known that in Europe general paralysis was not commonly reported until after the late seventeenth century […] It is therefore probable that among Europeans, it took many years for a treponeme variety with a nervous affinity to be created through adaptation and selection. The tropical races thus find themselves at the same stage as the whites of Europe were a few centuries ago, when, as in the tropics today, the pox was exceptionally serious but had no later impact on the central nervous system.^29^

The neurotropic strain was thus thought to be the product of civilisation, while the ‘primitive’ nature of the colonised peoples had enabled the preservation of an original, more benign form of syphilis. The common association of ‘civilisation’ and ‘syphilisation’ was thus seen not only in the growing spread of syphilis in the colonies but also in the development of a more dangerous version of the disease, similar to that seen among Europeans, among the ‘natives’.

Following subsequent research, Levaditi and Marie confirmed the duality of the dermotropic and neurotropic ‘viruses’ in 1923, but further complicated the picture by stating that both were actually families of treponemes combining ‘numerous varieties endowed with specific biological properties, unequal virulence, and varying organotropism.’^30^ While some French doctors were convinced by these theories, they were by no means unanimously accepted. A number of physicians with significant experience in the colonies opposed the dualist hypothesis in the 1920s and 1930s, including Georges Lacapère,^31^ Albert Sézary and Marcel Léger. Sézary, a dermatologist and syphilographer who had done his medical training in Algiers, was one of the most outspoken critics of Levaditi and Marie’s work, which he attacked in two articles published in the early 1920s.^32^ In the 1923 article, he criticised what he called the ‘neurotropic virus doctrine’ as being unfounded. In Sézary’s view, it was not a matter of the nature of the treponeme but of the degree of civilisation of those affected. In his opinion, the colonised populations represented ‘uncivilised peoples, whose nervous systems are not overexerted’, whereas general paralysis and tabes dorsalis only affected ‘individuals who have been initiated into modern civilisation’.^33^ This idea was supported by a fact unanimously recognised by French dermatologists and syphilographers during the interwar period: the increase in the number of cases of nervous forms of syphilis among certain colonised populations in particularly close contact with ‘civilisation’, notably in North Africa and Indochina.

Sézary’s thinking evolved over the course of the next decade, to the point where he doubted the very existence of ‘exotic syphilis’ as it had been thus far conceived. In 1932, he published a long article that struck down most of the theories that had been proposed to explain it in prior decades: different infectious agents, the influence of race, the introduction of alcohol, early contamination^34^ and the role of civilisation. This included a rejection of the hypothesis he had proposed in 1923: ‘we do not believe that the role of civilisation, considered as a factor of cerebral overexertion, merits the importance it has been accorded’.^35^ It was primarily Sézary’s study of French rural populations in the 1920s that convinced him there was no link between civilisation and general paralysis, since he found that the inhabitants of the countryside, although less cerebrally overexerted than those in urban areas, were no less affected by nervous forms of syphilis: ‘country people are no more immune to this complication than city dwellers’. This comparison between the colonised and rural populations was an increasingly common feature of the colonial medicine discourse on syphilis during the interwar period.

Challenges to theories positing the existence of variants of syphilis became more common in the 1930s. Marcel Léger, a doctor who had worked in various posts in French West Africa, Tonkin, the French West Indies and Marseille, insisted for example in 1931 that syphilis was a cosmopolitan disease: ‘There is no metropolitan syphilis and colonial syphilis, a European syphilis and an exotic syphilis; there is only one infection caused by the same pathogen, Schaudinn’s treponeme’.^36^ Nevertheless, the idea that the syphilis among colonised populations was less dangerous than that contracted by Europeans remained largely dominant until the mid-twentieth century. Sézary himself supported the discourse about the relative benignity of syphilis among the ‘natives’. He also agreed that there had been an increase in the number of cases of tabes dorsalis and general paralysis in the colonies but saw this as the consequence of an entirely different phenomenon: the introduction of European therapies.

## Mass treatment with *blanchiment*

During the interwar years, the venereal disease control armamentarium largely consisted of mercury, bismuth and most importantly arsenic derivatives. Arsenobenzenes in particular were very widely used and favoured by doctors. In 1917, for example, one year into the French occupation of Cameroon, the favoured treatment for syphilitic patients in the hospital in Douala was ‘606’ (Salvarsan) or ‘914’ (Neosalvarsan). In a report on the early days of AMI, the director of the Cameroon Health Service waxed lyrical about the success of these therapies and the satisfaction he claimed to see among patients:

Arsenobenzol has an excellent effect on full-blown syphilis and the blacks are very satisfied with the intravenous medication, which improves their general condition very soon after the initial dose and leads to the rapid recession of their symptoms.^37^

Stocks of these drugs were however often limited and shortages were common, notably due to the high cost of arsenobenzenes. Colonial physicians therefore continued to use bismuth and mercury salts throughout the 1920s and 1930s, as revealed by the annual reports of the French West Africa Health Service.^38^ The decision to use bismuth on economic grounds reflected a common phenomenon in Africa, as seen in the treatment of both syphilis and certain tropical endemic diseases such as yaws, for example in the Belgian Congo, Rwanda and Kenya.^39^

Syphilis treatment was provided via the existing public health system in each colony of the French empire. In most of the territories, this was characterised by highly unequal provision between urban and rural settings. Urban centres, which were few in number, generally had hospitals, maternity units and relatively extensive dispensary networks (although there were significant differences between the colonies: Dakar, where an Institute of Social Hygiene, the Roume Polyclinic, was built in 1931, was by far the best equipped urban centre in French West Africa, of which it was the administrative capital). Conversely, the rural areas in which the majority of the population lived were served only by a very small number of dispensaries covering a tiny portion of the territory. In some regions, these dispensaries were supplemented by mobile units. Sometimes, as in Cameroon and Togo, the duties of the mobile teams that had previously specialised in sleeping sickness control were expanded to cover the prevention of other diseases.

While venereal disease control strategies varied from one colony to another, certain practices were common across the empire, such as the introduction of the regulation of prostitution.^40^ Another common practice was *blanchiment*, the use of which has been confirmed in both Africa and Indochina and which was the subject of a broader debate about how to treat the colonised populations. In simple, generic terms, *blanchiment* consisted of giving patients partial, superficial treatment with the acceptance that this would not cure them completely – i.e. that it would not eliminate the infectious agent (or at least try to do so). For French colonial doctors and authorities, this practice had two advantages. Firstly, it provided the illusion of a cure, as a few injections of novarsenobenzol or bismuth produced the disappearance of the external, skin symptoms of the disease – without removing the treponemes from the body. Secondly, it ‘sterilised’ patients for a certain period of time – a few weeks or months – or in other words made them temporarily non-contagious. The practice was therefore primarily one of mass preventive medicine, drawing its legitimacy from the idea that *blanchiment* would reduce new infections and therefore the spread of the disease and forming part of the genealogy of Treatment as Prevention (TasP).^41^

This medical approach, based on cutting the chains of contamination, was neither new nor unique to syphilis in the colonial context. Similar methods, aimed primarily at stopping transmission rather than curing patients, were used to control other tropical endemic diseases in colonised Africa.^42^ As early as 1906, the use of Atoxyl during the sleeping sickness campaign in French Equatorial Africa was based on the idea of reducing the risk of infection.^43^ In the case of yaws, Dawson has also analysed the debates surrounding preparations for mass campaigns in Kenya in the 1920s. Of the various solutions that were considered, the one that was chosen by the British Medical Department was ‘to render a large proportion of the population non-infectious by means of injections in order to prevent new cases’. The use of new treatments for the rapid disappearance of skin symptoms made this option particularly attractive to medical officers: ‘Early on physicians realized that the new arsenic-based drugs “magically” seemed to cleard up secondary yaws lesions’.^44^ Under a variety of names and forms, *blanchiment* was a widely used method for the control of colonial endemics.

During the interwar period, in the case of syphilis, this method was primarily employed in rural dispensaries in the French colonies. To a lesser extent, *blanchiment* was also practised by the AMI mobile teams after the expansion of their activities in the 1930s. This was the case in Cameroon, where it was presented as the most effective form of action, as in this 1936 Health Service report:

Syphilis prevention is done much more profitably, without significant laboratory resources, by the mobile teams. They may not cure every patient, but through their ‘mass’ action, and through the mandated gathering of the entire population,^45^ and the large number of personnel, they rapidly implement the *blanchiment* of entire sectors, thus reducing the circulating virus, and enabling viable infants to be conceived.^46^

The desire to move to a more active form of syphilis screening and treatment in the colonies was primarily motivated by concerns about the birth rate and ‘racial preservation’, i.e. the need to safeguard future generations from a ‘degeneration’ that would harm the development and exploitation of the overseas territories. These concerns had already motivated a differentiated approach to the treatment of syphilis among the colonised populations, with pregnant women and infants receiving ‘thorough’ treatment (i.e. a complete cure) in maternity units in urban centres.^47^ The dispensaries, however, especially those in rural areas, were limited to providing incomplete treatment. This was partly due to the fact that most of the patients who consulted these services were male^48^ and therefore not the primary target of venereal disease control efforts. Maternity units also provided a way to monitor women with syphilis to ensure that they completed the course of treatment.^49^ In this context, the deployment of mobile teams was primarily seen as a way of reaching a wider audience of women who were pregnant or of childbearing age. Because the maternity units only covered a tiny part of the population, the *blanchiment* of future mothers was seen as an effective, economical practice outside urban centres. During the 1930s, a number of colonial physicians in Africa also advocated the systematic roll-out of mobile teams specifically targeting syphilis, modelled on the approach to sleeping sickness control introduced by the *Mission Jamot.*

## The debate about *blanchiment* in the 1930s

During the interwar period, *blanchiment* was widely used to treat syphilis in the colonies. Some physicians did, however, begin to develop doubts about the method, questioning its effectiveness or raising concerns about potential iatrogenic effects. Controversy broke out over the practice in the early 1930s, at a time when the ‘success’ of the Jamot method was a source of genuine enthusiasm among some French dermatologists and syphilographers, who hoped to apply this model to venereal disease control.

One of the main instigators of this controversy was Marcel Léger, who consciously opened up the debate with a paper given to the Société de Pathologie Exotique [Society of Exotic Pathology] on 13 May 1931.^50^ In it, he condemned the excessive use of arsenobenzenes in the colonies, which he argued was responsible for the increase in cases of tabes dorsalis and general paralysis among the colonised peoples. He emphasised that syphilis was not a skin disease but a ‘general’ disease whose skin manifestations were only one of several others.^51^ Léger thus directly refuted the hypothesis of an exotic, dermotropic *Treponema pallidum* that, by downplaying the seriousness of syphilis among the colonised populations, had helped to justify the use of *blanchiment.* Less than a month later, on 10 June 1931, he published a new communication in which he was openly critical of the difference between syphilis treatment among the European and colonised populations:

there can be no such thing as syphilis treatment specific to the inhabitants of hot countries. If precautions must be taken, and there are dangers to be avoided in relation to Whites, then we should strive to take these same precautions, and avoid these same dangers, when dealing with those with Blacks or Yellows. Syphilis is a cosmopolitan social disease. No matter in what country we find ourselves, we must not conceive of any intentional under-medication or any medication that could lead to serious illness in the long term.^52^

In addition to the fundamental neglect represented by only partially treating patients with syphilis, Léger also pointed out two major limitations of *blanchiment:* its ineffectiveness and its iatrogenic effects. With regard to the former, he observed that treated individuals quickly became contagious again, after just a few months, while the removal of the external signs of the disease would facilitate its transmission.^53^ With regard to the latter, he identified European therapies as playing a role in the increase of cases of general paralysis and tabes dorsalis among the ‘native’ populations. It was this concern, rather than questions about the effectiveness of *blanchiment,* that would go on to become the focus of debate during the 1930s.

Since the early twentieth century, the idea that nervous forms of syphilis (general paralysis and tabes dorsalis) were on the rise in certain parts of the world, where the disease had previously presented solely with skin manifestations, had gained ground. Jeanselme had earlier described this phenomenon in Japan, attributing it to the country’s gradual adoption of a European lifestyle.^54^ From 1910–20 onwards, there was a surge in reports of the emergence or increase of nervous forms of syphilis. Most doctors subscribed to the idea of a change taking place in the majority of colonies but agreed that it was still very limited. Other than the dualist hypothesis of the existence of both dermotropic and neurotropic ‘viruses’, this phenomenon was generally explained by the rising degree of civilisation, the introduction of alcohol or the spread of the neurotropic form of the virus. In the early 1930s, however, a new angle on the question emerged: what if European therapies were the primary cause?

This idea was analysed in detail by Sézary in an article published in 1932 in the *Annales de dermatologie et syphiligraphie* [Annals of Dermatology and Syphilography],^55^ one of France’s leading syphilography journals. He began by confirming the observation that cases of tabes dorsalis and general paralysis were on the rise in the colonies – ‘it cannot be denied that a change has occurred in recent decades: nervous syphilis, although still not very widespread among the exotic peoples, is becoming less exceptional there’^56^ – which he had reportedly noticed himself during this time in Algeria. After ruling out the main theories previously developed to explain this phenomenon – including multiple forms of the infectious agent, race, changes in behaviour, a higher degree of civilisation and the introduction of alcohol – Sézary homed in on one factor that he believed explained both (1) the differences observed in the symptoms between Europeans and the ‘natives’ and (2) the increase in nervous forms of syphilis among the latter: European methods of treatment. This realisation had come upon him gradually. In 1926, he had believed that it was the length of time syphilis had been present in a society that explained the rise in nervous forms of the disease, via the development of a kind of imperfect immunity limiting the skin symptoms.^57^ But in the intervening years, he had abandoned this idea: ‘We are now persuaded […] that it is the therapeutic methods developed by the civilised peoples that have been the main cause, both in Europe and throughout the world, of the changes that have occurred in the symptomatology of syphilis.’^58^

Sézary drew on the history of syphilis in Europe to support his argument, claiming that nervous involvement was rare until the nineteenth century and that the disease had previously presented with skin manifestations similar to those seen in the colonial populations. He argued that the shift began with the use of mercury, before being further accelerated by the introduction of arsenic derivative therapies,^59^ and that the same shift had begun to take place among the colonised peoples since the introduction of European therapies.^60^ He did not hold the drugs themselves responsible but rather the fact that the treatment of patients with syphilis was very often ‘insufficient’, i.e. incomplete. According to Sézary, ‘insufficient treatment at the primary-secondary stage can only be harmful’ and could even be ‘more harmful than abstinence from treatment’ because it deprived the body of ‘its natural defences’.^61^ This view directly echoed the representation of ‘exotic syphilis’ as a benign disease by suggesting that skin manifestations had a protective effect against the ‘more serious’ nervous forms. The phenomenon described by Sézary, i.e. the fact that treatment – if insufficient – caused general paralysis and tabes dorsalis, could supposedly be seen at the individual level^62^ but even more so at the collective level, because the tendency to nervous forms was passed on in a hereditary manner: ‘This movement must be considered in the community and across the generations of that community. We believe that the current neurotropism of the disease has been created by our insufficient medications having blocked the body’s immunisation efforts over several generations’.^63^ ‘Exotic syphilis’ was thus presented as an original form of the disease, in the sense that its manifestations were those that had not been exacerbated by the use of European therapies.

During the 1930s, while opinions remained divided between the dualist thesis and the iatrogenic hypothesis,^64^ it was the latter that became the focus of the debate about *blanchiment.* The idea that insufficient treatment would result in the transformation of ‘exotic syphilis’ (i.e. that seen among Africans or Asians), which primarily manifested in skin and bone involvement, into a less benign disease attacking the nervous system, called into question the colonial methods of syphilis control. The most stringent criticism came from members of the Institut Prophylactique [Prophylactic Institute] of Paris, which played a major role in venereal disease control in France during the interwar period.^65^ The institution advocated providing all patients with personalised treatment until they had been fully cured, as confirmed by serology testing.^66^ This approach to the treatment of syphilis was incompatible with the colonial practice of *blanchiment.* The general application of the Institut Prophylactique’s methods to the colonies was, however, considered utopian by a large majority of doctors, as it not only would require huge human and financial resources^67^ but also would take years, if not decades, to make it accessible to everyone living in the empire.^68^ The syphilographers of the Institut Prophylactique acknowledged that it would have to be a long-term endeavour but maintained that *blanchiment* with arsenobenzenes should be proscribed due to its iatrogenic impact. In their view, the most reasonable solution would be either to use other less effective, but also less iatrogenic, drugs such as mercury or pentavalent arsenic^69^ or to ‘abstain from treatment’ and let syphilis continue to develop in ‘benign’ skin forms.^70^

## The rhetoric of neglect

Criticisms of *blanchiment* were generally poorly received in the French colonial medicine sphere. Of the physicians who accepted the idea that superficial treatment was responsible for the development of more severe nervous forms of syphilis in the colonies,^71^ a majority still remained in favour of this approach, except in urban centres where comprehensive treatment was felt to be a possibility. The first to speak out after Léger presented his article on 13 May 1931^72^ was none other than Eugène Jamot, who supported *blanchiment* on the grounds that it brought about a temporary break in transmission. In Cameroon, the colonised populations were subjected to a ‘standard treatment’ protocol consisting of five injections of novarsenobenzol: ‘under its influence, the contagious lesions disappear and after *blanchiment* the patient remains harmless for several months, and often much longer.’^73^ He also argued that even if *blanchiment* was harmful to some patients, it was of benefit to the majority: ‘It may well be that this system is ultimately harmful to a few patients, but the question is whether in order to spare a few individuals we should leave an entire population exposed to infection.’^74^ This conception of mass colonial medicine in the treatment of syphilis was consistent with the approach that Jamot had taken to sleeping sickness control.

Jamot’s view of *blanchiment* was widely shared among colonial physicians, all of whom saw the collective benefit as overriding the risk of individual harm. The iatrogenic effects were fully accepted, for example by Dr Botreau-Roussel:

By administering these therapies, even if they are somewhat irrational and incomplete, we are at least providing preventive social medicine, and in the end what does it matter if we cannot, with these standard treatments, prevent a few syphilitic patients from developing tabes dorsalis or general paralysis, if they have prevented them from contaminating hundreds, if not thousands of others.^75^

In the colonial world, however, this logic applied only to the colonised populations, because the colonists themselves received personalised treatment designed to bring about a total cure.^76^ Sézary himself also defended the practice of *blanchiment*, arguing that ‘the first task of social medicine must be to eliminate the viral reservoirs.’^77^ In his view, the risks of individuals developing general paralysis and tabes dorsalis, as well as that of the disease evolving – according to his own theory – into a less benign form in the future, simply had to be accepted. His article responded directly to Léger’s criticisms. For Sézary, the solution lay instead in adapting what he saw as a method with proven effectiveness to treat syphilis: ‘It may be a good idea to draw inspiration from the system of flying units that has been so successful in French West Africa in the fight against sleeping sickness.’^78^

The moral dimension of *blanchiment* was an integral part of the debate. This was routinely introduced in the form of a dilemma, presented in such a way that it left little room for criticism: should ‘native’ syphilitic patients undergo *blanchiment* or be left to their fate? Five months after Léger’s first paper was presented to the Société de Pathologie Exotique, S. Golovine (stationed in Dakar) responded to him in the society’s meeting on 14 October 1931.^79^ He presented *blanchiment* as the only possible course of action, at least in the colony with which he was familiar, Senegal. He also posed the question in terms that left little room for discussion: ‘Should we treat syphilitics in our little bush stations, or abandon them completely to their fate, making no attempt at all to combat this colonial scourge?’ This rhetoric helped to justify *blanchiment* by reducing the debate to a choice between ‘treating’ or ‘abandoning’ patients. This presented it as the moral duty of colonial doctors to use the resources at their disposal to treat ‘native’ patients with syphilis, in a context in which ‘sadly, “blanchiment” is as things stand *currently the only weapon* we have in this fight!’^80^ Golovine felt that physicians had a moral duty to reduce infections through *blanchiment* and called on his colleagues to join him: ‘We can at least say to ourselves in all conscience: *feci quod putus; faciunt meliora potentes.’* ^81^ In the discussion that followed the paper, Léger launched a scathing attack on what he saw as the wholly deluded advocates of *blanchiment* with arsenobenzenes: ‘It’s like a fireman pouring a little water on a fire with a child’s bucket and then retreating into the distance. Because he can no longer see the fire, he concludes that he has put it out.’^82^ Until complete treatment of all patients in the colonies was possible, he supported either the use of mercury or abstinence from treatment.

Ultimately, the rhetoric designed to justify the practice of *blanchiment* was always founded on the argument of insufficient resources. A majority of doctors had a highly fatalistic attitude about their capacity to do more under the conditions in which they practised and with the resources available to them, particularly in the bush or in rural areas. This lack of material and human resources was the basis for the argument that there was no alternative to *blanchiment* other than to abandon patients to their fate. But over and above the lack of available resources, there were concerns about the cost of syphilis control. This was indeed a major drain on the supply budgets of the colonial health services. In Cameroon in 1936, the joint syphilis and yaws control campaign used the equivalent of 812 000 francs worth of drugs (arsenic derivatives, bismuth and mercury), equating to one third of the total value (approximately 2 450 000 francs) of the drugs distributed in the colony over the year.^83^ In comparison, the sleeping sickness control campaign was much less costly (about 500 000 francs). In the Health Service annual report for 1936, Médecin-Colonel Lefèvre also emphasised the value of *blanchiment* in terms of resource management: ‘In our opinion, this is the best way, given our present resources, to provide a thorough, effective medical service without unnecessarily wasting specific medicines.’^84^ The lack of resources that was used to justify *blanchiment* was down to budgetary decisions made by the colonial administration. The supply budget for Cameroon was drastically cut in 1936, by order of the Commissioner of the Republic. In his report, Lefèvre was very clear about the consequences of these budget cuts: ‘there is no doubt that we will find it very difficult to treat patients in 1937’.^85^

The drive to cut costs largely prevailed over public health concerns, with some doctors openly expressing their view that the costs of venereal disease control had to be reduced at the expense of its effectiveness. One of the options considered was to limit the use of arsenobenzenes in favour of very low-cost mercury therapies. Supporters of this approach included Médecin Général F. Sorel, former director of the French West Africa Health Service, who stated in 1933:

In the long term, there is no medical service budget that can bear the cost of all the arsenobenzol or bismuth compounds necessary to treat the multitude of ailing blacks: some individuals may, I am aware, find themselves less well off, but if we recognise that it is not possible to make an exception for such patients by giving them the new products, I believe it is preferable to sacrifice a few individual interests for the greater good of the community.^86^

This analysis reveals the full ambiguity of the colonial discourse about the impossible nature of the fight against venereal diseases. While continuing to insist that *blanchiment* was the only solution given the resources currently allocated, the performance of medical action justified the withdrawal of investment from an item of expenditure deemed to be particularly costly.

## Colonial fatalism and medical racism

While the argument of insufficient resources was central to the rhetoric used to justify *blanchiment*, this fatalistic attitude to material questions was paralleled by a fatalistic attitude among doctors founded on racial prejudices. This was primarily expressed in two forms: (1) identifying the ‘natives’ as being those primarily responsible for the failures of French medical efforts due to their ‘lack of care’ and ‘neglect’^87^ and (2) presenting the situation as being impossible to improve in the short term due to their insufficient degree of civilisation. Naturally, these two arguments were woven together in a medical discourse that condemned the limited adherence of African or Asian populations to the – brutal – enterprises of colonial medicine.

(1) The first dimension was both a racial prejudice and a common feature of the medical literature, in Health Service reports and in the official discourse of the colonial administration. According to the French, even if sufficient resources were allocated, it would still be impossible to cure the colonised populations because of their non-adherence to treatment over the long term. Doctors criticised patients for no longer attending the dispensaries for once the external symptoms of the disease had disappeared. In this narrative, *blanchiment* was no longer a deliberate enterprise by colonial physicians but a consequence of the ‘lack of care’ and ‘neglect’ of the patients themselves. This point of view was clearly expressed by health service officers, such as the author of the 1935 medical report on the Ivory Coast, who stated, ‘The natives are negligent, syphilis accidents go unnoticed by them and women are reluctant to consult for this type of disease’^88^, or the Médecin-Colonel Lefèvre, who argued that doctors had given up on complete treatment because they were ‘discouraged’ by the ‘lack of care’ shown by the Cameroonians.^89^ For colonial doctors this ‘lack of care’ was expressed in various forms, including discontinuing treatment after *blanchiment*, careless sexual relations that would inevitably lead to reinfection and ignorance of the disease and its manifestations. This final point was highlighted by Jamot in 1931: ‘For the native, syphilis is a superficial disease and very often, when after two or three injections the lesions that led him to seek treatment have disappeared, he believes himself to be cured and leaves the hospital.’^90^ The idea of ‘neglect’ was a common feature of the colonial discourse. Doctors associated it with the fact that people did not take care to consult a doctor when they developed the first symptoms of the disease and continued to have sex despite their infection. Here, the inconsistency of the colonial medicine discourse in its attempt to justify *blanchiment* is striking: despite the broad consensus that ‘exotic syphilis’ was essentially a benign skin disease, this rhetoric held the ‘native’ populations themselves responsible for downplaying the danger of the disease.

This idea was accompanied by the argument that it was the ‘natives’ themselves who were demanding arsenobenzene-based therapies and driving up their use in the colonies. This was frequently asserted by colonial doctors and administrators, further strengthening the narrative that *blanchiment* was a consequence of the behaviour of the colonised populations themselves, and also featured in the reports issued to the League of Nations in the context of administration of the mandated territories. According to the author of the 1923 report for Cameroon, for example, ‘the natives are besieging the medical stations to obtain the wonder drug’.^91^ Charles Massias also supported this discourse that held the colonised peoples responsible for both *blanchiment* and the spread of the disease, observing with regard to the people of Indochina that, ‘An increasing number of syphilitics are clamouring to be treated with novarsenobenzol. But most of the time, the Annamites only agree to receive a few injections’.^92^ Excessive or incorrect use of arsenic derivates was also therefore blamed on the colonised populations. Few doctors failed to succumb to this convenient discourse, but Léger, again in opposition to most of his colleagues, criticised the argument for its inconsistency: ‘doctors must guide their patients and not allow them to demand a particular medication. Do they mindlessly give morphine to everyone who asks for it?’^93^

(2) The racist views of doctors and the colonial administration resulted in a fatalistic attitude to the fight against venereal diseases in the colonies, which was thought to be a losing battle due to the ‘primitive’ mindset and the inadequate degree of ‘civilisation’ among their inhabitants. Any form of additional investment was thought to be useless under present circumstances, on the grounds that such people were unable to properly look after themselves. This was one of the angles from which Golovine defended *blanchiment* in 1931:

*We cannot sit idly by*, waiting for syphilimetry to be made available across the overseas possessions and for the social education of the savage peoples to be raised to the right level at which complete treatment of syphilis can be achieved.^94^

This view not only highlighted the legitimacy of colonisation and its ‘civilising mission’ but also justified a certain neglect in venereal disease control. Before investing further resources, the populations must be ‘educated’ and made to understand the benefits of medical modernity brought to them by the coloniser. Because adults were, however, considered impossible to educate in such a way, this would have to be done by the French school system. Teachers were thus turned into the guarantors of the future health of the societies under domination. In Cameroon, in 1917, ‘a series of elementary lessons that have been copied and translated into the local language’ were distributed to all primary schools. In them, Dr Martin, director of the Health Service, stated that ‘since the Blacks are precocious, we have included a few words about venereal diseases.’^95^

During the interwar years, doctors continually presented the social level of the colonised populations as being insufficient to support effective venereal disease control. This observation not only absolved the colonial power of its failures in this area but also justified its limited investment. The prospect of abandoning *blanchiment* in favour of comprehensive treatment was continually put off to an unspecified later date.^96^

## Conclusion

The neglect of syphilis in the French colonial empire, during the interwar period, was the product of a complex interplay of scientific knowledge, medical practices, racial conceptions and colonial policies. In the colonies, syphilis was presented as a benign ‘skin disease’, except for the European population. By inventing an ‘exotic syphilis’, colonial medicine was able to draw a dividing line between its practices and those carried out in the context of venereal disease control in metropolitan France. Once the consequences of the disease had been downplayed and the inability of the ‘natives’ to adhere to treatment was accepted, *blanchiment* appeared to be an acceptable practice. By performing the (bio)medical modernity placed at the service of the colonies and their inhabitants, this mass treatment combined the illusion of effectiveness with a pragmatism designed to limit the cost of venereal disease control. Insufficient resources were presented as an inevitability by the colonial doctors and used to justify *blanchiment.* But it was the fatalistic discourse of colonial medicine itself that maintained the shortage. The argument that it was impossible to treat the colonised populations properly, due to their behaviour and degree of civilisation, was a justification for the saving of resources in the treatment of syphilis.

With the economic crisis of the 1930s, the colonial government’s veiled withdrawal of investment in venereal disease control went even further. A 1940 report on Togo, for example, shows that the number of syphilis-related consultations fell by more than 75% in 1935, following a ‘reorganisation’ of the health services.^97^ This trend reflected a move to impose budget restrictions across the empire. It did not go unnoticed. In early 1935, at the Conférence économique métropolitaine et coloniale [Metropolitan and Colonial Economic Conference], the Social Welfare Committee expressed concern about the cuts: ‘The Committee observed a regrettable tendency to shift some of the impact of the crisis onto the health services and to reduce their resources. […] It judged that a period of economic crisis was, on the contrary, an opportune moment to take advantage of the slowdown in work to strengthen health policy and to place the population in the best possible conditions of health and strength.’^98^ Economic challenges made it even harder for the few opponents to the mass use of *blanchiment* for treating syphilis patients in the colonies to make themselves heard. The alternative advocated by the doctors of the Institut Prophylactique – a full course of treatment accompanied by close serological monitoring over several months – would have required huge, large-scale investment. The racial, medical and economic fatalism that prevailed in the years leading up to the Second World War exerted far too great an influence for any course to be considered other than neglect of the ‘venereal peril.’

## Footnotes

1 On sleeping sickness control campaigns and the *Mission Jamot*, see Guillaume Lachenal , ‘Médecine, comparaisons et échanges inter-impériaux dans le mandat camerounais: une histoire croisée franco-allemande de la mission Jamot’, Canadian Bulletin of Medical History, 30, 2 (2013), 23–45 2815539910.3138/cbmh.30.2.23; Jean-Pierre Dozon , ‘Quand les Pastoriens traquaient la maladie du sommeil’, Sciences Sociales et Santé, III, 3–4 (1985), 27–56 11635938; Jean-Paul Bado , Eugène Jamot, 1879-1937: Le médecin de la maladie du sommeil ou trypanosomiase (Paris: Karthala, 2011).

2
Virginie De Luca Barrusse, ‘Natalisme et hygiénisme en France de 1900 à 1940. L’exemple de la lutte antivénérienne’, Population, 64, 3 (2009), 531–60; Virginie De Luca Barrusse *Population en danger! La lutte contre les fléaux sociaux sous la Troisième République* (Bern: Peter Lang, 2013).

3 This was the idea that syphilis could be inherited from a syphilitic parent, even beyond the first generation: Alain Corbin, ‘L’hérédosyphilis ou l’impossible rédemption. Contribution à l’histoire de l’hérédité morbide’, *Romantisme*, 31 (1981), 131–‑50; Claude Quétel, *Le mal de Naples* (Paris: Seghers, 1986), 208–-21.

4 Myron Echenberg, ‘“Faire du nègre”: military aspects of population planning in French West Africa, 1920-1940’, in D.D. Cordell and J.W. Gregory (eds), *African Population and Capitalism: Historical Perspectives* (Boulder: Westview Press, 1987); Delphine Peiretti-Courtis, *Corps noirs et médecins blancs. La fabrique du préjugé racial, XIX^e^-XX^e^ siècles* (Paris: La Découverte, 2021), 234–52.

5 On French colonial medicine, see in particular Aly Pam Adama, *Colonisation et santé au Sénégal (1816-1960)* (Paris: L’Harmattan, 2018); Laurence Monnais, *Médecine et colonisation. L’aventure indochinoise, 1860-1939* (Paris: CNRS, 1999); Guillaume Lachenal, *Le médicament qui devait sauver l’Afrique. Un scandale pharmaceutique aux colonies* (Paris: La Découverte, 2014); Myron J. Echenberg, *Black Death, White Medicine: Bubonic Plague and the Politics of Public Health in Colonial Senegal, 1914-1945* (Portsmouth, NH: Heinemann; Oxford: James Currey, 2002); Rita Headrick, *Colonialism, Health and Illness in French Equatorial Africa, 1885–1935* (Atlanta: African Studies Association Press, 1994); Claire Fredj, ‘Le laboratoire et le bled. L’Institut Pasteur d’Alger et les médecins de colonisation dans la lutte contre le paludisme (1904-1939)’, *Dynamis*, 36, 2 (2016), 293–316.

6

Megan Vaughan , ‘Syphilis in Colonial East and Central Africa, the social construction of an epidemic’, in T. Ranger and P. Slack (eds), Epidemics and Ideas: Essays on the Historical Perception of Pestilence (Cambridge: Cambridge University Press, 1992), 269–302 ; Megan Vaughan , Curing Their Ills: Colonial Power and African Illness (Stanford: Stanford University Press, 1992), in particular 129–53. See also, for example, Maryinez Lyons , ‘Sexually Transmitted Diseases in the History of Uganda’, Genitourinary Medicine, 70 (1994), 138–45820647510.1136/sti.70.2.138PMC1195212; Bryan T. Callahan , Syphilis and Civilization: A Social and Cultural History of Sexually Transmitted Disease in Colonial Zambia and Zimbabwe (Baltimore: John Hopkins University, 2002).

7

João Dinis Sousa , Philip J. Havik and Anne-Mieke Vandamme , ‘Sexually Transmitted Infections, their Treatment and Urban Change in Colonial Leopoldville, 1910–1960’, Medical History, 65, 2 (2021), 178–96; Philip J. Havik , ‘Public Health, Social Medicine and Disease Control: Medical Services, Maternal Care and Sexually Transmitted Diseases in Former Portuguese West Africa (1920–63)’, Medical History, 62, 4 (2018), 485–506 3019178410.1017/mdh.2018.44PMC6158648; Daniel J. Walter , Sex and Control: Venereal Disease, Colonial Physicians and Indigenous Agency in German Colonialism, 1884-1914 (New York: Berghahn, 2015).

8

Idrissou Alioum , ‘Prostitution et lutte contre les maladies vénériennes au Cameroun, 1923-1964: discours et pratiques’, Kaliao, 2, 4 (2010), 123–41; Yacouba Banhoro , ‘Essai historique sur la prostitution et la prophylaxie antivénérienne dans la Haute-Volta coloniale’, Annales de l’Université de Ouagadougou, 20, A (2015), 179–212 . On North Africa, see also Hannah-Louise Clark , ‘Civilization and Syphilization: A Doctor and His Disease in Colonial Morocco’, Bulletin of the History of Medicine, 87, 1 (2013), 86–114 2360353010.1353/bhm.2013.0003.

9 Kalala Ngalamulume, ‘Le péril vénérien. L’Etat colonial français et la sexualité à Saint-Louis du Sénégal, 1850-1920’, in J.-P. Bado (ed.), *Les conquêtes de la médecine moderne en Afrique* (Paris: Karthala, 2006); Mor Ndao, ‘Les prises en charge des pathologies vénériennes par le pouvoir colonial au Sénégal, 1850-1960. Éclairages et enseignements pour le sida’, *Annales de la Faculté des Lettres et Science Humaines,* 39, B (2009), 40–68; Charles Becker and René Collignon, ‘A history of sexually transmitted diseases and AIDS in Senegal: difficulties in accounting for social logics in health policy’, in P. W. Setel, M. Lewis and M. Lyons (eds) *Histories of Sexually Transmitted Diseases and HIV/AIDS in Sub-Saharan Africa* (Westport, CT: Greenwood, 1999), 65–96.

10 On the challenges posed by skin NTDs today and the work being done by the World Health Organization to control them, see *Ending the Neglect to Attain the Sustainable Development Goals: A Strategic Framework for Integrated Control and Management of Skin-Related Neglected Tropical Diseases* (Geneva: World Health Organization, 2022).

11 For a discussion of the need to consider history in NTDs research, see Guillaume Lachenal *et al.*, ‘Neglected Actors in Neglected Tropical Diseases Research: Historical Perspectives on Health Workers and Contemporary Buruli Ulcer Research in Ayos, Cameroon’, *PLOS Neglected Tropical Diseases*, 10, 4 (2016), e0004488.

12 On the interconnected issues of race and medicine in colonised Africa, see in particular Vaughan, *op. cit.* (note 6); Peiretti-Courtis, *op. cit.* (note 4); Karen Jochelson, *The Colour of Disease: Syphilis and Racism in South Africa, 1880–1950* (New York/Oxford: Palgrave/St Anthony’s, 2001).

13 Edouard Jeanselme, ‘La syphilis dans la péninsule Indo-Chinoise’, *Annales de dermatologie et de syphiligraphie*, ser 4, II (1901), 817–37.

14 These lectures were transcribed and published in 1904 as Édouard Jeanselme, *Cours de dermatologie exotique*, collected and edited by M. Trémolières (Paris: Masson & cie, 1904).

15 Including, for example, Georges Lacapère, a venereologist who worked in Morocco and wrote *La Syphilis arabe: Maroc, Algérie, Tunisie* (Paris: Doin, 1923). See Clark, *op. cit.* (note 8), 101.

16 Jeanselme, *op. cit.* (note 14), 135–57.

17
*Ibid.*, 135.

18
*Ibid.*, 136.

19
*Ibid.*, 138.

20
*Ibid.*, 146.

21
*Ibid.*, 157.

22

Katherine Paugh , ‘Yaws, Syphilis, Sexuality, and the Circulation of Medical Knowledge in the British Caribbean and the Atlantic World’, Bulletin of the History of Medicine, 88, 2 (2014), 243 10.1353/bhm.2014.002924976161.

23 Samuël Coghe, *Population Politics in the Tropics: Demography, Health and Transimperialism in Colonial Angola* (Cambridge: Cambridge University Press, 2022), 168–70; Shane Doyle, *Crisis and Decline in Bunyoro Population and Environment in Western Uganda 1860-1955* (Oxford, Kampala and Athens, OH: James Currey, Fountain Publishers and Ohio University Press, 2006), 150–7; Headrick, *op. cit.* (note 5), 36–41.

24

Hideyo Noguchi and Joseph W. Moore , ‘A Demonstration of Treponema Pallidum in the Brain in Cases of General Paralysis’, The Journal of Experimental Medicine, 17, 2 (1913), 232–81986764010.1084/jem.17.2.232PMC2125022.

25 Constantin Levaditi and Auguste Marie (de Villejuif), ‘Le tréponème de la paralysie générale’, *Comptes rendus de l’Académie des sciences* CLVIII (1914), 1593–5.

26

Constantin Levaditi and Auguste Marie , ‘Etude sur le tréponème de la paralysie générale’, Annales de l’Institut Pasteur, 33, 11 (1919), 741–76.

27 Two human subjects, working on experiments with rabbits and monkeys, were inoculated with syphilis as part of this work: the first accidentally and the second voluntarily.

28 Levaditi and Marie, *op. cit.* (note 26), 750–1.

29
*Ibid.*, 751.

30

Constantin Levaditi and Auguste Marie , ‘La pluralité des virus syphilitiques’, Annales de l’Institut Pasteur, 37, 2 (1923), 190 .

31 Clark, *op. cit.* (note 8), 101–2. Georges Lacapère opposed the dualist theory in his book *La Syphilis arabe*, published in 1923: Lacapère, *op. cit.* (note 15).

32 Albert Sézary, ‘Pathogénie de la paralysie générale et du tabes’, *Revue neurologique,* 4 (1921), 338–47; Albert Sézary, ‘La pathogénie des affections nerveuses parasyphilitiques et la doctrine du virus neurotrope’, *Revue neurologique*, II, 5 (1923), 414–23. Levaditi and Marie had responded to Sézary’s first publication in their article published in early 1923: Levaditi and Marie, *op. cit.* (note 30).

33 Sézary, ‘La pathogénie des affections’, *op. cit.* (note 32), 418.

34 Some doctors explained the lack of progression to nervous forms of syphilis among Arab, African and Asian populations as being due to the fact that they supposedly had their first sexual relations at an earlier age than Europeans.

35

Albert Sézary , ‘Le problème de la syphilis exotique. Pathogénie et thérapeutique’, Annales de dermatologie et de syphiligraphie, 7, III (1932), 981–3.

36

Marcel Léger , ‘Considérations sur le traitement de la syphilis chez les Indigènes de nos colonies’, Bulletins de la Société de pathologie exotique et de ses filiales de l’Ouest africain et de Madagascar, 24 (1931), 434 .

37 M.G. Martin, ‘Les premiers résultats de l’Assistance Médicale aux Indigènes du territoire occupé de l’ancien Cameroun’, Douala, 28 February 1917; ANOM (Archives Nationales d’Outre-Mer) 1 AFFPOL 3237, 12.

38 For the period 1920–39, see ANS (Archives Nationales du Sénégal) 2G 20, no. 15; 2G 21, no. 17; 2G 22, no. 18–20; 2G 23, no. 26–7 and 33; 2G 24, no. 25–8; 2G 25, no. 27; 2G 28, no. 17–27; 2G 29, no. 20–32; 2G 30, no. 14–23; 2G 31, no. 21–30; 2G 32, no. 26–35; 2G 33, no. 17–25; 2G 34, no. 16–26; 2G 35, no. 15–24 and 28–35; 2G 36, no. 20–44 and 34–40; 2G 37, no. 21–30; 2G 38, no. 11–22; 2G 39, no. 9–15 and 24–7. See also the annual reports for French West Africa (AOF), archived at ANOM: GUERNUT 51 (reports 1931–5), GUERNUT 58 (1936) and AGEFOM 383 (1935–9).

39

Anne Cornet , Politiques de santé et contrôle social au Rwanda: 1920-1940 (Paris: Karthala, 2011), 174–80. See also Mark H. Dawson , ‘The 1920s Anti-Yaws Campaigns and Colonial Medical Policy in Kenya’, International Journal of African Historical Studies, 20, 3 (1987), 427 11617206.

40 In sub-Saharan Africa, these were largely ineffective due to the inability of the French colonial authorities to understand the cultural practices of African societies: Alioum, *op. cit.* (note 8), 131–6; Banhoro, *op. cit.* (note 8).

41 Guillaume Lachenal, ‘A genealogy of treatment as prevention (TasP): prevention, therapy, and the tensions of public health in African history’, in T. Giles-Vernick and James L.A. Webb Jr. (eds), *Global Health in Africa: Historical Perspectives on Disease Control* (Athens, OH: Ohio University Press, 2013), 70–91.

42 Headrick, *op. cit.* (note 5).

43 Deborah Neill, ‘Paul Ehrlich’s Colonial Connections: Scientific Networks and Sleeping Sickness Drug Therapy Research, 1900–1914’, *Social History of Medicine*, 22, 1 (2009), 70. For more on the history of the trypanosomiasis control campaigns, see also Samuël Coghe , ‘Sleeping Sickness Control and the Transnational Politics of Mass Chemoprophylaxis in Portuguese Colonial Africa’, Portuguese Studies Review, 25, 1 (2017), 57–89 ; Coghe, *op. cit.* (note 23); Dozon, *op. cit.* (note 1); Lachenal (notes 1 and 5); Noémi Tousignant, ‘Trypanosomes, Toxicity and Resistance: The Politics of Mass Therapy in French Colonial Africa’, *Social History of Medicine*, 25, 3 (2012), 625–43; Mari K. Webel , The Politics of Disease Control. Sleeping Sickness in Eastern Africa, 1890–1920 (Athens, OH: Ohio University Press, 2019).

44 Dawson, *op. cit.* (note 39), 420–1.

45 This term is underlined in the source material.

46 Health Service annual report. Cameroon (1936); ANOM GUERNUT 58, 245.

47 Health Service annual report. Cameroon (1936); ANOM GUERNUT 58, 222.

48 This phenomenon was reported by most bush doctors in sub-Saharan Africa in the Health Service reports, for example in Côte d’Ivoire: annual medical report (1928), ANS 2G 28-18, 38; Inspection report (27 March to 2 May 1934), ANS 1H26 (AOF), 33; annual Health Service report (1934), ANS 2G 34, no. 23, 47. In Senegal: Health Service annual report (1934), ANS 2G 31, no. 30, 50–1; in Cameroon: ANOM 1 AFFPOL 3237, 12 (1917); *Rapport sur l’administration sous mandat des territoires du Cameroun (1923)* (Paris: Imp. Générale Lahure, 1924), 59–60.

49 On the regulation of maternity in the French colonial empire, see Claire Fredj, ‘Encadrer la naissance dans l’Algérie coloniale. Personnels de santé et assistance à la mère et à l’enfant “indigènes” (XIX^e^-début du XX^e^ siècle)’, *Annales de démographie historique*, 122, 2 (2011), 169–203.

50 Marcel Léger, ‘Les arsénobenzènes aux Colonies: leur emploi raisonné et dûment contrôlé’, *Bulletins de la Société de pathologie exotique et de ses filiales de l’Ouest africain et de Madagascar*, 24 (1931), 394–406.

51
*Ibid.*, 395.

52 Léger, *op. cit.* (note 36), 434.

53
*Ibid.*, 435.

54 Jeanselme, *op. cit.* (note 14), 146–7.

55 Sézary, *op. cit.* (note 35).

56
*Ibid.*, 978.

57

Albert Sézary , ‘Syphilis exotique et pathogénie de la syphilis nerveuse’, La presse médicale, 34, 1 (1926), 6.

58 Sézary, *op. cit.* (note 35), 988.

59 Albert Sézary and Mlle. Roudinesco, ‘L’incubation du tabès et de la paralysie générale’, *Bulletins et mémoires de la Société médicale des Hôpitaux de Paris*, ser. 3, 47, 19 (1931), 968–77.

60 Sézary, *op. cit.* (note 35), 988–90.

61
*Ibid.*, 992.

62 It was thought that an under-treated patient would develop a nervous form more frequently and more rapidly than an untreated patient. Similarly, a syphilis patient insufficiently treated with arsenic derivatives was thought to develop paralysis or tabes dorsalis more rapidly than one treated with mercury: Sézary and Roudinesco, *op. cit.* (note 60).

63 Sézary, *op. cit.* (note 35), 993.

64 Proponents of the dualist thesis included, for example: M.A. Gauducheau, ‘Syphilis exotique et tabès’, *Bulletin de la Société de pathologie exotique et de ses filiales de l’Ouest africain et de Madagascar*, 25 (1932), 680–5; Constantin Levaditi, ‘Étiologie et mécanisme pathogénique de la parasyphilis’, *La prophylaxie antivénérienne*, 7, 1 (1935), 133–61.

65 The Institut Prophylactique was founded in 1916 by the syphilographer Arthur Vernes: Gérard Tilles , ‘En finir avec la syphilis. L’ambition inaboutie d’Arthur Vernes’, Annales de dermatologie et de vénéréologie, 147, 1 (2020), 57–73 3186476310.1016/j.annder.2019.11.003.

66 A patient was considered to be cured after eight successive monthly tests came back negative.

67

S. Golovine , ‘Observations sur le traitement de la syphilis aux colonies’, Bulletins de la Société de pathologie exotique et de ses filiales de l’Ouest africain et de Madagascar, 24 (1931), 639–44.

68 In 1935, some twenty centres or laboratories affiliated to the Institut Prophylactique were established in the French colonies. ‘Service de l’Institut Prophylactique’, *Archives de l’Institut Prophylactique*, VII, 1 (1935), 9–10.

69 Léger, *op. cit.* (note 36), 438. See also F. Sorel, ‘La syphilis chez les indigènes en Afrique Occidentale’, *Archives de l’Institut Prophylactique*, V, 1 (1933), 23–4.

70 This option was proposed in particular by Charles Massias: ‘La syphilis nerveuse chez les indigènes d’Indochine, syphilis dite exotique et manifestation nerveuses’, *Annales de dermatologie et de syphiligraphie,* ser. 7, VI (1935), 99–100.

71 Some doctors did, however, reject this hypothesis, such as Mme Delanoë: ‘Au sujet de l’emploi des arsénobenzènes au cours du traitement de la syphilis indigène’, *Bulletins de la Société de pathologie exotique et de ses filiales de l’Ouest africain et de Madagascar*, 24 (1931), 369–71.

72

Marcel Léger , ‘Les arsénobenzènes aux Colonies: leur emploi raisonné et dûment contrôlé’, Bulletins de la Société de pathologie exotique et de ses filiales de l’Ouest africain et de Madagascar, 24 (1931), 394–406 .

73 Eugène Jamot in Léger, *op cit.* (note 51), 404. The doses of novarsenobenzol were increased over the course of the treatment, from an initial dose of 0.45g to the final two doses of 0.90g.

74
*Ibid.*, 405.

75 Léger, *op. cit.* (note 36), 439.

76 Racial distinctions in treatment were the norm. This was still the case in the 1950s, in the context of the sleeping sickness control campaigns: Lachenal, *op. cit.* (note 5), 115–24.

77 Sézary, *op. cit.* (note 35), 996.

78
*Ibid.*, 997.

79 A sub-heading under the title of his article reads, ‘Regarding the articles published by M. Marcel Léger’: Golovine, *op. cit.* (note 67).

80
*Ibid.*, 639.

81
*Ibid.*, 642–3.

82
*Ibid.*, 643.

83 Health Service annual report. Cameroon (1936); ANOM GUERNUT 58, 14.

84 Passage underlined in the original text: ANOM GUERNUT 58, 50.

85 A total of 1 691 000 francs was cut from the annual budget of the colony’s Health Service: ANOM GUERNUT 58, 17.

86 Sorel, *op. cit.* (note 70), 24.

87 The question of blaming and assigning responsibility for the production of diseases or the failure of public health action has been the subject of recent research. See, for example, Paul Farmer , Aids and Accusation: Haiti and the Geography of Blame (Oakland, CA: University of California Press, 2006); Samuel Kline Cohn , Epidemics: Hate and Compassion from the Plague of Athens to AIDS (Oxford: Oxford University Press, 2018); Paul-Arthur Tortosa, ‘Aetiology of Blame: Fevers, Environment, and Accountability in a War Context (France and Italy, c. 1800)’, *Centaurus. Journal of the European Society for the History of Science*, 65, 1 (2023), 63–90.

88 Health Service annual report. Ivory Coast. ANS 2G 35, no. 28, 37.

89 ANOM GUERNUT 58, 244–5.

90 In Léger, *op cit.* (note 51), 404.

91
*Rapport annuel sur l’administration sous mandat des territoires du Cameroun (1922)* (Paris: Imp. Générale Lahure, 1923), 37.

92 Massias, *op. cit.* (note 71), 98.

93 Léger, *op. cit.* (note 36), 436.

94 Golovine, *op. cit.* (note 68), 643.

95 Martin, *op. cit.* (note 37), 19.

96 The British colonial authorities and medical officers used the same justification in relation to the 1920s anti-yaws campaign in Kenya, presenting the prospect of raising standards of living and sanitation as a ‘utopian dream, prohibitively expensive as well as politically impossible’: Dawson, *op. cit.* (note 39), 420.

97 There were 35 698 consultations in 1933, 40 341 in 1934, 13 875 in 1935, 11 953 in 1936, 11 316 in 1937 and 9 647 in 1938. Secret report produced by Médecin Général Inspecteur Blanchard, ‘L’œuvre sanitaire de la France au Togo’, 1 November 1940, Clermont-Ferrand, 16-17. ANOM AFFPOL 358.

98 ‘Travaux de Commission de la prévoyance sociale, Conférence économique métropolitaine et coloniale’, Algiers, 10 March 1935, 2–3. ANOM 100 APOM 187.

## Translator

Hayley Wood.

